# Clinical Management and Neurosurgical Approach of Pott’s Puffy Tumor: A Case Report

**DOI:** 10.7759/cureus.63686

**Published:** 2024-07-02

**Authors:** Leopoldo Mandic Ferreira Furtado, Luiza Cassino Gil Nunes, Cristina Gabriela Fernandes, Patricia Cruz Guimarães Pinto, José Aloysio Da Costa Val Filho

**Affiliations:** 1 Pediatric Neurosurgery, Hospital Vila da Serra/Oncoclínicas, Nova Lima, BRA; 2 Pediatrics, Hospital Vila da Serra/Oncoclínicas, Nova Lima, BRA

**Keywords:** pediatric neurosurgery, acute sinusitis, intracranial epidural abscess, pott's puffy tumor, neuroinfection

## Abstract

Pott’s puffy tumors are assumed to be infrequent concomitant intra- and extracranial abscesses, mainly secondary to complicated frontal sinusitis during infancy. Due to the close proximity to the superior sagittal sinus, there is a risk of developing venous infections, thrombosis, and morbidity. In this case report, we present a case of an 11-year-old girl who presented with headache and face edema. After recognizing the Pott’s puffy tumor pattern on the CT scan and brain MRI, the neurosurgical approach involved pus evacuation and frontal sinus blockage, and the patient received antibiotic therapy and was evaluated for total recovery. To our knowledge, the prompt diagnosis and treatment of such conditions are paramount to avoid complications, and differential diagnosis should be encouraged in medical practice.

## Introduction

Pott’s puffy tumor (PPT), despite its name, is a non-neoplastic disease and is a subperiosteal and intracranial infection that was named by Percivall Pott, who, in 1760, described inflammation of the dura mater and skull with the formation of a mass in patients who have suffered brain trauma. Additionally, the “puffy” term in PPT refers to the soft appearance of the matter under the frontal skin [1,2].

Currently, this condition is defined as the collection of an abscess under the frontal pericranium associated with local osteomyelitis and an epidural abscess caused by direct implantation of bacteria secondary to sinusitis and dental infection or hematogenous spread [3,4]. Moreover, until 2006, only 37 cases were reported worldwide. More recently, 92 cases, including both children and adolescents, have been reported, as well as anecdotal reports in adults [1,4-6].

The potential for serious complications of PPT due to sagittal sinus thrombosis, cerebral vascular events, and meningitis is well known, and the need to indicate aggressive treatment as soon as possible since the diagnosis has been made has become urgent [7-11]. Therefore, the purpose of the present case report is to describe an unequivocal case of PPT, highlighting the main nuances of diagnosis, neurosurgical approach, and follow-up.

## Case presentation

History

An 11-year-old girl was admitted to the emergency room due to persistent headache for 12 days, associated with edema of the forehead. According to the patient’s parents, the condition has progressed quickly and the patient was evaluated for edema in the forehead and periorbital region 12 hours before ER presentation. Additionally, no skin color alterations on the forehead, vomiting, fever, or deterioration of consciousness or behavior were observed.

Furthermore, the patient’s parents reported three previous episodes of sinusitis in the last two years, and the last episode occurred 14 days before the aforementioned symptoms. They also mentioned that amoxicillin and clavulanate were administered for 10 days.

On physical examination, the child presented intact consciousness, as did her cranial nerves. Direct and indirect reactions of light to pupils were noted. No motor abnormalities were observed. In addition, forehead edema with extension to both periorbital regions was observed. There was pain on touch as well.

Investigations

**Figure 1:**
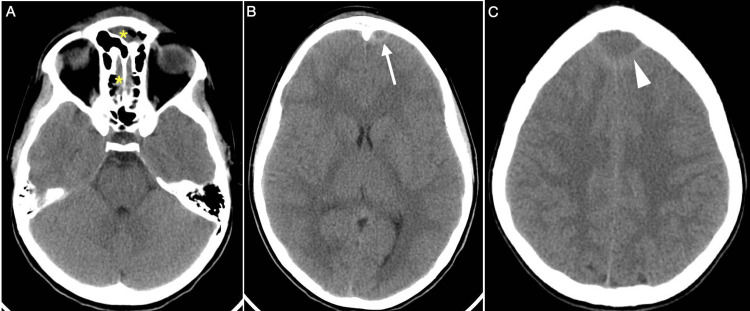
CT scan with no contrast of Pott’s puffy tumor. (A) A CT scan with no contrast displayed sinusitis of the ethmoidal and frontal sinuses (yellow asterisk). An epidural collection of pus is noted near the inner frontal protuberance (white arrow) and superior sagittal sinus (arrowhead) in images (B) and (C), respectively.

After the first clinical assessment, a CT scan of the head was taken, and a midline frontal epidural collection was obtained. The possibility of suppuration was considered (Figure 1).

Moreover, erosion of the inner layer of the frontal bone as well as a point of linkage to the extracranial bone was observed. The collection exerts a mild mass effect on the brain.

**Figure 2:**
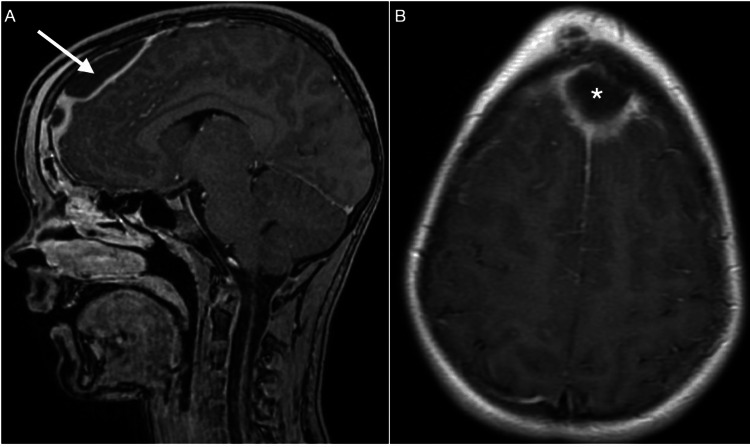
Preoperative MRI of Pott’s puffy tumor. Postgadolinium T1 MRI showing peripheral enhancement of the epidural collection exerting a mass effect on the frontal lobe in the sagittal slice (arrow) in (A), and proximity to the superior sagittal sinus is shown on the axial slice (asterisk) (B).

Due to the suspicion of intracranial epidural abscess, a brain MRI was carried out (Figure 2), which revealed an infection pattern in the collection; therefore, given the previous diagnosis of repeated sinusitis in combination with the clinical and radiological findings, the diagnosis of a Pott’s puffy tumor was confirmed.

Treatment

Initially, intravenous ceftriaxone was administered at a dosage of 100 mg/kg/day for one day, and the neurosurgical approach was carried out on the second day.

**Figure 3:**
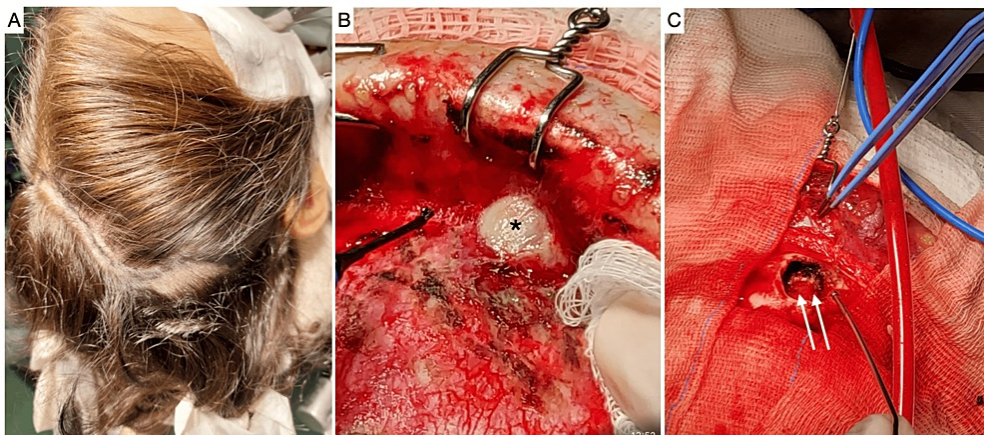
Neurosurgical approach of Pott’s puffy tumor. The bicoronal incision marked with minimal hair shaving is shown in image (A). After the removal of the skin flap, a pus collection is observed emerging to the pericranium (asterisk) (B), and the resulting hole after pus removal of the epidural space and surroundings as well as bone irrigation is depicted in image (C) (double arrows).

During the surgery, a bicoronal incision was made, followed by pericranium removal, and suppurative fluid was identified and collected for examination. The compromised frontal bone was enlarged using a drill, and the epidural space was assessed and irrigated with saline solution. A microscope was used to inspect the epidural space. Additionally, the infected mucous in the frontal sinus was removed, and the frontal sinus was subjected to electrocautery to remove the infected tissue. Normal pericranium was used to block the frontal sinus and to avoid recurrence. Separated stitches were applied at the end of the surgery (Figure 3).

Follow-up

**Figure 4:**
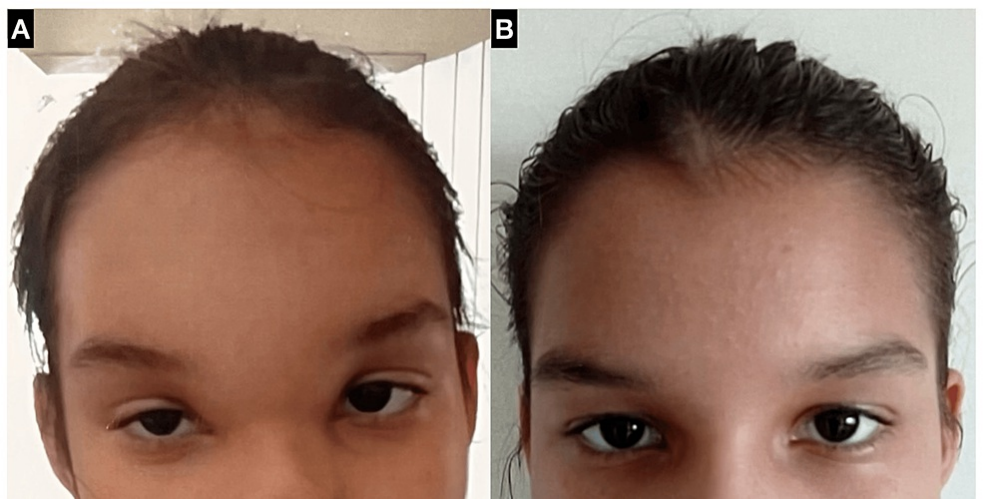
Physical examination of Pott’s puffy tumor before and after treatment. The edema of the forehead and orbits seen during the active infection (A) was completely resolved during the first month of treatment (B).

During the uneventful postoperative period, the headache improved, and the edema decreased completely after 20 days (Figure 4).

Discharge from the hospital was achieved after 40 days of intravenous treatment with ceftriaxone 2g 12/12 hours, vancomycin 500 mg 6/6 hours, and metronidazole 500 mg 8/8 hours and the symptoms were completely resolved.

**Figure 5:**
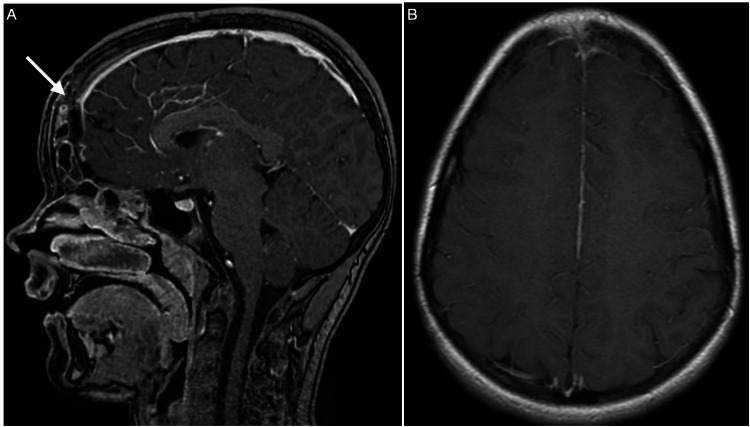
Postoperative MRI findings after Pott’s puffy tumor treatment. Six-month MRI showing the epidural collection had completely drained, and the patency of the superior sagittal sinus remained. Small bone continuity is observed (arrow) (A), and frontal convexity is regular in shape (B).

**Figure 6:**
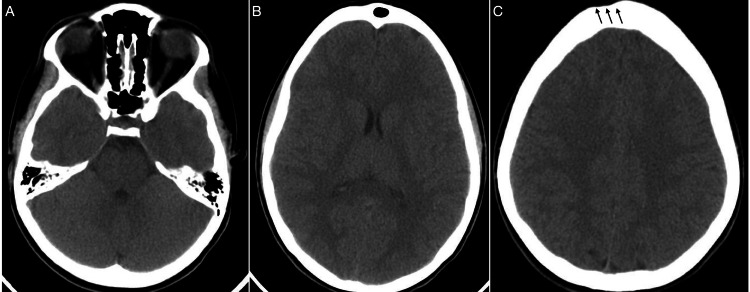
CT scan with no contrast after the treatment of Pott’s puffy tumor. CT scan after one year of treatment showed the absence of sinusitis with regular pneumatization of the frontal and ethmoidal sinuses (A). Expansion of the frontal lobe near the inner frontal protuberance (B) and a tiny depression on the surface of the frontal bone (triple black arrows) (C).

Six months later, a new MRI revealed the resolution of intracranial abnormalities (Figure 5), and this pattern remained even after one year, as shown on the CT scan (Figure 6).

## Discussion

Although several publications have indicated that PPT is a rare condition, the incidence of this condition has increased worldwide, mainly owing to rhinosinusitis, head trauma, odontogenic infection, substance use, and even COVID-19 and mucormycosis [4,11-13].

Although infections such as sinusitis and otitis are relatively common during infancy, a minority of cases are complicated with intracranial suppurative collections, including brain abscess, subdural empyema, and epidural empyema [14,15]. On the other hand, because pneumatization of the frontal sinus usually occurs during adolescence, PPT is more common at this time, with the typical presentation of fluctuating edema of the forehead that progresses to the orbits. Rarely, the internal compartment of orbits can be compromised [16].

In the present case, the suppurative collection on the intracranial compartment was confirmed by CT scan and brain MRI, thanks to the interdisciplinary team composed of pediatricians and pediatric neurosurgery acknowledging this condition, as has been pointed out in the literature for the success of early diagnosis and treatment [17]. Furthermore, the main risk during management is the initiation of antibiotic treatment with no surgical evacuation and the evaluation of thrombophlebitis of the superior sagittal sinus and its blockage with venous infarction of the brain, and even subdural empyema, pyogenic meningitis, and brain abscess [18]. Another risk is orbital extension of pus and progression to vision impairment. In such cases, an ophthalmologic assessment is also important [16].

To evacuate and depurate the infection, an open surgical approach with minimal craniectomy or an endonasal approach with frontal and ethmoidal sinus clearance could be performed, depending on the expertise of the service [1]. To our knowledge, the upper approach has the advantages of releasing pressure from the superior sagittal sinus via suppurative collection, clearing direct vision, and blocking communication between the frontal sinus content and epidural space. During surgery, incidental durotomy must be avoided due to the risk of dissemination of infection to the leptomeninges and the brain.

## Conclusions

The early diagnosis of PPT as well as the indication of an open neurosurgical approach in combination with the right choice of antibiotic therapy warranty not only the complete treatment and improvement of symptoms but also avoid relapses and morbidity.

Given that children who present with clinical signs of PPT could be misdiagnosed with skin infections, such as cellulitis, leading to neglect of intracranial suppuration, physicians should always bear in mind to take into account this differential diagnosis and conduct imaging investigations of the skull as soon as possible to avoid complications such as thrombophlebitis of the superior sagittal sinus. Therefore, the prompt recognition of this clinical entity is paramount to ensure early diagnosis and efficient treatment.
